# Changing the landscape of mental health among college students: a community case study of a course on learning sustainable well-being

**DOI:** 10.3389/fpubh.2023.1175594

**Published:** 2023-07-27

**Authors:** Karen Dobkins, Janna Dickenson, Debra Lindsay, Taylor Bondi

**Affiliations:** ^1^Human Experience and Awareness Laboratory (HEALab), Department of Psychology, University of California San Diego, San Diego, CA, United States; ^2^Sexual Well-being and Gender (SWAG) Lab, Department of Psychology, University of California San Diego, San Diego, CA, United States

**Keywords:** mental health, mindfulness, compassion, student well-being, resilience, sustainability

## Abstract

Our society is facing an unprecedented mental health crisis, with nearly one in two people being affected by mental health issues over their lifespan. This trend is especially noticeable among college students, who undergo significant shifts in social, familial, and academic responsibilities. Exacerbating the mental health crisis is the fact that students are facing other societal crises (e.g., climate change). And, in a reciprocal fashion, students experiencing poor mental health are less likely to feel resilient enough to tackle these other crises. In response to these colliding societal crises, we need a comprehensive solution that goes beyond the current models of college mental health services. We propose an alternative *preventative mental health* approach, which aims to prevent the onset of mental health concerns and build resilience in the face of colliding crises. Specifically, we argue that colleges can aid in building mental health resilience by creating for-credit courses that teach students the skills they need to be conscious, responsible, and resilient human beings. Toward this end, we created an experiential, workshop-style, 1 unit, P/NP course, entitled “Learning Sustainable well-being” (LSW), which guides students to explore, improve, and sustain their mental health. The principles taught in this course combine the wisdoms of several disciplines, including mindfulness, psychology, neuroscience, philosophy, religion, poetry, and cinema. The following community case study reflects on the journey of our “LSW initiative,” starting from the creation of the course in 2014 to the current mission of scaling up the offering as part of an institution-wide LSW program. To this end, we describe the LSW course modules/content, our pedagogical approach, potential limitations, and then provide data demonstrating its efficacy in improving student well-being. As a final note, we present the challenges we have faced, and the lessons learned, while on this journey. We hope that presenting this community case study will facilitate the growing dialogue across colleges about creating (and perhaps requiring) courses like LSW in order to improve students’ mental health and resilience in the context of other colliding crises.

## 1. Introduction

We are amid an unprecedented mental health crisis. Almost half of the people living in the US deal with mental health issues at some point in their lives (1–4). Although this trend is visible across all populations, college students represent an especially vulnerable community. During the college years, many young adults undergo significant shifts in their social, familial, and academic responsibilities. Such increases in social and academic demands introduce new stressors that carry the potential to burden students’ mental health. As such, the onset and prevalence of mental health disorders has been reported to peak during the college years (5–7). Moreover, college students report high levels of sub-clinical mental health symptoms. For example, a survey from 102 universities across the US (*n* = 103,748) revealed that a substantial proportion of students report high levels of loneliness (28%), anxiety (34%), depression (41%), and suicidal thoughts (13%). Additionally, 50% would like help with their mental health, whereas only 38% believe they are currently flourishing (8).

The recognition that colleges should provide students with any services beyond academic development started in the US about 150 years ago. In the early 1800s, colleges focused on promoting *physical* health, speculating that this had direct effects on academic performance. Many colleges implemented health courses, focusing largely on hygiene in an effort to prevent diseases and public health outbreaks, and by 1861, Amherst College had developed the first comprehensive health program [for a review, see (9)]. Since then, most colleges offer several services/resources for maintaining good physical health, both in terms of prevention (e.g., fitness facilities, physical education courses, and wellness programs) and treatment of disease (e.g., student health centers).

Approximately 50 years after the introduction of physical health services, colleges considered the importance of providing resources for student *mental* health [for a review, see (10)]. In 1910, Princeton began offering mental health services after observing that many well-qualified students were withdrawing from their studies citing “emotional problems.” While a movement arose to institute these mental health services, only a handful of other prestigious colleges followed suit over the next 10–15 years. During this time, university administration considered having a few part-time psychiatrists or counselors on staff to be sufficient. Shortly thereafter, World War II shed light on the impact of mental health. WWII soldiers returned to civilian life with “combat exhaustion”, which spurred a new category of diagnoses known as posttraumatic stress disorders and highlighted the need for a more wide-spread and comprehensive mental health movement. By the 1950s, mental health services and resources emerged throughout the US, including within most colleges (11). Today, colleges recognize the need to provide services and resources for student mental health, though the size and scope of these offerings varies by budget and resources (12).

While it is encouraging that most colleges in the US provide mental health services, these programs have several limitations. *First*, they are often under-staffed and over-burdened with administrative responsibilities, and therefore under-resourced to meet the high demand of students in need of treatment (13, 14). *Second,* mental health services tend to focus on treating concerns that meet DSM criteria, and as such, are utilized by students when problems have already become overwhelming. *Third*, and related to the last point, there are many students experiencing poor mental health (e.g., depression, anxiety, and loneliness) who do not consider themselves in need of mental health services and/or do not realize the benefits of partaking in practices that improve well-being. These students often report feeling that their problems are “not severe enough” to seek mental health services (8). *Fourth*, many students may choose to not seek help from mental health services for either practical reasons (e.g., lack of insurance coverage or perceived lack of time) or the fear of being labeled (or thinking of themselves) as having a mental “disorder.” This can be particularly salient for *underrepresented* students [see (15)], who sometimes report feeling that they do not belong and/or that their mental health symptoms will not be believed by staff (16).

All these limitations are compounded by the fact that we are amid other societal crises, with one example being climate change. A recent study conducted by Cambridge Global Perspectives (17) surveyed over 11,000 young people (ages 13–19) in multiple countries and found that over a quarter of them (26%) believe the climate crisis is the biggest issue facing the world today (39% of the US sample). Furthermore, the majority of the sample (92%) report having already changed their behavior because of the climate crisis. With the rise of the youth climate movement (18) it is clear that concerns about the climate crisis are an *additional* mental health burden for college students. While it is encouraging that college students have a heightened awareness of the climate crisis, this concern can lead to feelings of depression and helplessness. In fact, two recent studies have reported that exposure to direct outcomes of the climate crisis (e.g., extreme weather events) as well as indirect exposure through media reports, elevates risk of depressive, anxious, and posttraumatic stress symptoms (19, 20). Not only does this “climate-anxiety” add to the already existing mental health crisis, in a reciprocal fashion, people experiencing mental health challenges are less likely to feel resilient enough to tackle the climate crisis. Therefore, enhancing mental well-being among college students is necessary—not only to change the landscape of the mental health crisis but to also bolster students’ psychological aptitude to address other societal problems.

To address the compounding issues that threaten student mental health, some colleges have attempted to *expand* their mental health resources, for example, by offering group therapy and workshops, creating websites containing internal and external mental health resources, and providing free access to self-help and/or meditation apps (e.g., Headspace) [see (21, 22) for further examples]. Still, there is a growing gap between the needs of students and the resources being provided to them (12). As an example, students at our university often report that the decentralized nature of these varied resources leaves them feeling overwhelmed, a phenomenon referred to as the “tyranny of choice” (23).

In this paper, we propose a *different type* of resource for student mental health, which is *integrated* within their (very familiar) college experience of enrolling in courses. Specifically, we argue that colleges should offer for-credit “sustainable well-being” courses, where students learn the skills they need to be conscious, responsible, and resilient human beings. This for-credit course approach targeting mental well-being overcomes many of the limitations of the existing mental health services (outlined above), as well as providing additional benefits. *First* (and most importantly), well-being courses take a *preventative* mental health approach; rather than waiting for situations to reach a point where they are overwhelming, students can learn the skills to prevent those situations from escalating. *Second*, well-being courses can reach many students, as well as a diverse range of students. This includes students who: (1) currently feel that they are not facing challenging situations, (2) believe their problems are not severe enough to seek mental health services, (3) belong to minority groups that are typically underserved by mental health services. As an added benefit, taking a well-being course along with peers is likely to lower the stigmatization of mental health issues, as students get to see that almost everyone suffers from time to time. *Third*, because well-being courses are able to reach many students at once, the likely long-term consequence will be to lower the burden on student counseling centers, with the added benefit of being cost-effective for the institution. *Finally*, because these well-being courses are taught by professors, connection and community is built between professors and students, which will likely enhance the campus culture.

## 2. Providing context: a comprehensive solution for well-being courses at UCSD

To pave the path for academia to address colliding crises through for-credit course offerings, it is worthwhile to start by providing a bit of history about this journey at our own institution: the University of California, San Diego (UCSD). In 2003, the University of California system adopted a freshman seminar program, in which faculty are incentivized (with $1,000) to teach a small (20-person), low workload (1-unit, P/NP, 1 h/week), fun/engaging course on any topic to incoming freshman. The program, which is now popular on colleges across the country, was created in response to a growing student need to experience a more *intimate* learning environment, in contrast to most of their other courses where the large enrollments (300–400 students) can be de-personalizing and overwhelming. In 2014, the first author (Dobkins, a professor of psychology) created a freshman seminar, entitled “Learning Sustainable Well-being” (LSW), taught in an experiential, work-shop style format, with the goal of teaching students how to build healthy relationships with oneself and others.

After receiving feedback from many students that this was the most important course they had ever taken, it became clear that this course had the potential to change lives. In response to this, in 2019, the first author started a grass roots “LSW initiative” at UCSD, with the goal of expanding the LSW offering to more students. As such, the course was expanded to accommodate 100 students across all year levels and bring in four to five undergraduates (who had previously taken the course) to assist in facilitation of the exercises. (In addition, the weekly meeting time was increased from 60 to 80 min). In 2021, the second author (Dickenson, also a psychology professor) joined the LSW initiative, teaching her own section of the course as she and the first author worked together to improve the curriculum. Survey data collected since 2019 provides evidence that the course improves well-being, and testimonials reveal themes that emerged after taking the course (see “Data Showing Efficacy of the LSW Course,” *below*). In addition, many students report spreading the lessons from the course to their roommates and friends, which enhanced connections and improved campus culture.

Given the impact of the LSW course, it is now our long-term goal to create an official, and integrated, LSW *program* at our college. Our vision for this is twofold. *First*, we plan to recruit faculty from *other* departments to be trained in, and then teach, the LSW course. Faculty will be incentivized with monetary compensation as this 1-unit course is taught above current teaching load (current load being anywhere between 12 and 16 units/year, depending on the department). It is our hope that compensation will come from the institution, as is the case for freshman seminars, although we recognize the potential need to apply for outside funding. Recruiting professors from other departments is not only necessary to expand the course beyond our psychology department, but it also allows the teaching of the curriculum through other lenses, e.g., whereas psychologists might teach about anxiety in terms of the body’s fight or flight mechanisms, historians might reference time periods where a society was challenged with, and had to overcome, disasters (e.g., the creation of the Truth and Reconciliation Commission after the end of Apartheid).

*Second*, we plan for the LSW program to provide a *series* of course offerings, each focusing on a certain aspect of the human experience (and being subtitled accordingly). The current LSW course taught by Dobkins/Dickenson is subtitled: “Compassion for Self and Others,” as it is designed to help students explore, improve, and sustain their relationship with self and others. Next, the plan is to develop a course that helps students improve their relationship with the *environment*, with a particular emphasis on the climate crisis. Such efforts are currently underway at our college, spearheaded by Dr. Adam Aron. In sum, in response to the growing mental health crisis and the impact of other societal crises, we need a comprehensive solution; an LSW program that provides experiential learning on compassion and mental well-being, as well as courses on other colliding crises, for example: climate change, racial sensitivity, health disparities, or any other challenge facing society. Below, we discuss the elements of the current LSW course, which is focused exclusively on enhancing well-being through compassion for self and others.

## 3. Elements of the current LSW course headings

Below we provide information about A) the LSW course itself (description, pedagogical approach, and modules/content); B) potential limitations of the course; and C) data showing its efficacy in improving well-being.

### 3.1. Course description, pedagogical approach, modules, and limitations

#### 3.1.1. Course description

The principles taught in the current “LSW: Compassion for Self and Others” course combine the wisdoms of several disciplines, including mindfulness, psychology, neuroscience, philosophy, religion, poetry, and cinema, which are drawn from a large time span (500 BC to the present day). Each week, there is a short lecture on a given topic, combined with workshop-style exercises. The exercises include: (1) private reflection; (2) group discussion; (3) didactic discourse between the instructor and students; and (4) partnering up (students taking turns facilitating each other on an exercise). After each class, students are sent follow-up announcements with additional resources such as podcasts, blogs, and vlogs. Passing the course requires simply (1) attending the course in person (students are allowed to miss 1 of the 10 meetings and can make-up any missed class by attending office a hours); (2) submitting a weekly reflection essay describing what they got most out of the class for that week; and (3) a final reflection video summarizing their overall experience in the course.

#### 3.1.2. Pedagogical approach

The course was designed with the philosophy that students learn best through demonstrations. As such, the course was designed to be taught in a *demonstrative* fashion, with the instructor “acting” out vignettes of human life, from own or others’ lived experiences. Here, the word “demonstrative” is meant to have two different, but related, meanings. In the conventional sense, demonstrative means to openly show one’s emotions, with the effect that the instructor self-discloses in front of the students (while maintaining healthy boundaries). In a less conventional sense, the word demonstrative refers to the act of demonstrating, with the goal of others then being able to mimic the process, much like a yoga teacher demonstrating a pose for participants to follow.

#### 3.1.3. Course modules/content

The course has 10 modules (each 80 min), one for each week of the quarter. This translates to 13.33 h of class time. In addition, weekly reflections (approximately 15 min/week) and optional material for students (approximately 15 min/week) may add another 5 h of outside class time over the quarter. For each module, we provide a brief outline of the concepts taught, and one or two examples of in-class exercises.

Week 1: Practicing Psychological Well-being: Insights from Eastern and Western Philosophies.

Concepts:

- How humans evolved into beings who suffer psychologically
- Practices/wisdoms for alleviating suffering, based on both Eastern and Western approaches
  - Exercises:
- “Share your Voice”—Randomly pop up and say “why I am taking this course”
- “Wiggle it Out”—Leave the class dancing to music

PART 1: SELF-COMPASSION: RELATIONSHIP WITH SELF.

Week 2: Exploration of the Self, and How to Live a Life with Heart.

Concepts:

- Historical perspective of the “Self” from philosophers, old and new, (from John Locke, William James, Alan Watts and Sam Harris)
- Gaining awareness of negative self-talk, and changing your relationship to it
- Learning to honor the layers of self, from the core (innermost) to the persona (outermost) layer
- Behaving in alignment with core values and following a path with heart
- Learning to succeed means learning from, and being okay with, your failures

Exercises:

- “Mindfulness Meditation”—Practice two different types of meditation, and write down what you heard your mind say
- “Core Values Journaling”—Write down your core values, and notice what aspects of persona you are attached to

Week 3: Accepting All of Your Personality Traits: the Good, the Bad and the Ugly.

Concepts:

- Connecting to our highest selves (i.e., desirable traits) while learning to forgive our lowest selves (i.e., undesirable traits)
- The shadow side of humans (from Carl Jung); a result of our creatureliness (from Sigmund Freud) and a need to make oneself important in the uncertainty of death (from Soren Kierkegaard)
- How to understand, and be gentle with, the shadow side of yourself, so that it does not lead to hurtful behaviors

Exercises:

- “Self-Love Meditation”—Holding oneself in the highest positive regard (from Carl Rogers)
- “Diffuse and Understand Negative Traits”—How is your negative trait a gift, or how does it *think* it’s serving you?

Week 4: Building Emotional Resilience by Challenging Your Thoughts and Changing Behaviors.

Concepts:

- What is emotional resilience? Perspectives from Mindfulness, Positive Psychology and Cognitive Therapy
- Learning to question what your “gremlins” are saying, and how to talk to your anxiety
- How to stop pretending and get in touch with the truth inside yourself

Exercises:

- “I Cannot Mind-Read”—Journal about a challenging time when you assumed you knew what someone was thinking/intending.
- “Discover the Raw Truth”—Go from the complicated story of something that troubles you to a one-line statement of the basic thought or emotion underlying it (e.g., “My friend getting married makes me sad because it reminds me that I am still single and not even close to getting married,” an example from the show “Friends”)

Week 5: Building Emotional Resilience by Accepting All of Your Emotions.

Concepts:

- Learning to notice, allow and accept, emotions …. in the body (from Tara Brach)
- Distinguishing unhealthy vs. healthy negative emotions (from Albert Ellis)

Exercises:

- “Body Scan” (Yoga Nidra)—A meditation on body parts.
- “RAIN”—Recognize, Allow, Investigate in a Non-judgmental and Nurturing way. Meditating on joy and pain in the body (from Tara Brach)

COMPASSION FOR OTHERS: RELATIONSHIP WITH OTHERS.

Week 6: Compassion for Others alongside Healthy Boundaries.

Concepts:

- “Being” with others, without trying to fix or change them (from Brené Brown, Marina Abromavic)
- Seeing the ways we separate from others through judgments, comparisons, and assumptions
- Setting healthy boundaries with others—you are not a mind reader, it’s not your job to fix people, everyone has their own reality

Exercises:

- “Just Like Me Meditation”—This person wishes to be happy, just like me
- “Are We Really That Different?” Think of a negative trait you do not like in others. What defense would you come up with to convince someone that—even though you might have this trait “a little bit”—it does not *really count*?

Week 7: Putting Compassion for Others into Practice.

Concepts:

- Shifting from judgment of, to compassion for, others
- Learning to see the “bully” as someone who needs help, not punishment (from Thích Nhất Hạnh)
- How to not take things personally

Exercises:

- “Eye Contact Exercise”—a joint meditation with another
- “Shifting from Judgment to Compassion”- Tell a story about someone who did something you did not like. First, from a position of judgment, then from a position of compassion (not pity) because you can relate to this person’s behavior.

Week 8: Approaching Conflict with Others from a “Needs” Perspective.

Concepts:

- Communicating needs without blaming others (from Abraham Maslow, Marshall Rosenberg)
- Learning to listen without defensiveness
- Shifting out of victim mode

Exercises:

- “Rumi Meditation”—Out beyond ideas of wrongdoing and rightdoing, there is a field, I will meet you there.
- “Knowing your Needs.” How can you ask for a need to be met (a) without labeling/making assumptions about the other person; and (b) without asking the other person to *feel* a certain way.

Week 9: Taking Responsibility for Conflict with Others.

Concepts:

- Taking responsibility for contribution to a conflict, no matter how small
- How to apologize and mean it!
- How to get honest with yourself about why another person triggers you

Exercises:

- “Shifting from Blame to Responsibility”- Tell a story about someone who you are having conflict with. First, from a position of blame, then from a position of taking responsibility (without putting yourself in the “doghouse”)
- “What is Actually Bothering You?”—When another person’s behavior has upset you, ask yourself (a) what story do you have about its significance? and (b) what are your actual concerns?

Week 10: Summary, Tips for Practicing.

Exercises:

• Participants come up and share their experiences and breakthroughs

#### 3.1.4. Limitations

Due to the nature of the LSW course, there is potential concern that students might enroll believing that the course will “fix their problems.” It is important to make clear to students (when they first enroll and throughout) that the course is not about fixing any specific problem, but rather, about learning a set of skills that can be applied to challenging situations, current and future. More specifically, the LSW course is not meant to address mental health *disorders*, acute concerns, or traumatic events, as these situations typically require a therapeutic approach. As such, if they are needed, students must be provided the resources on campus that provide such therapeutic assistance.

### 3.2. Data showing the need for, and efficacy of, the LSW course

Beginning in 2019, we started collecting data to (1) assess students’ need for an LSW course and (2) investigate whether students in the LSW course improve on several self-report measures of well-being. With regard to needs assessment, data collected across a wide swath of students who have not taken LSW (*n* = 6,051) show that when asked “how interested would you be in taking a well-being class if it counted toward your college requirements,” over 90% of students reported being interested, with the mode response being “extremely interested.”

With regard to improvements in well-being as a result of taking the LSW course, we collected measures right before the start of the quarter in which they took the course (referred to as the “pre-course” data) and then again at the beginning of the following quarter (referred to as the “post-course” data). Note that providing pre-course data was a requirement of the LSW course, whereas providing post-course data was voluntary yet incentivized with course credit in whatever course they were taking the following quarter (and thus, we were not able to obtain post-course data from all students). Collecting both the pre- and post-course data at the *beginning* of a quarter hopes to remove the effects of variation in well-being across the quarter (due to midterms, finals, etc.). However, because student well-being can change *between* quarters, it is important to have a control group as a comparison. Ideally, this control group would be a “wait-list” control, comprised of people who have signed up for the course, but are put on a wait-list to take the course later. This was not possible because, although the current LSW course always fills to capacity (currently, *n* = 65) with a wait-list, the number of students on the wait-list is typically small (15) because most students do not continue to add their name to a wait-list once that list is more than 25% of the course maximum. Instead, we took a different approach for recruiting control samples: other professors in our department who are teaching a psychology course in the same quarter as LSW were approached to recruit their students as a control sample. For those recruited students to be included in our control sample, they had to respond positively to a question that asks whether they would be interested in taking a well-being course at UCSD (as well as respond negatively to having previously taken LSW), and in this way, the control sample was matched in “interest in taking a well-being class” to the LSW sample.

To date, we have pre- and post-course data from 133 LSW students and 222 control students, collected over six academic quarters between 2019 and 2022. The sample was largely skewed toward women (80%), as is typically the case in Psychology departments. In addition, in three of the six quarters, the course was taught over Zoom, instead of in-person, because of COVID-19, with the result that 63.1% of the sample experienced the course in-person. In testing the effectiveness of the course on well-being, we additionally asked whether these demographics (gender or modality) affected the findings.

#### 3.2.1. Students’ overall experience

In post-course data, when LSW participants were asked about their overall experience, 97% percent said they either agreed or strongly agreed that the LSW course improved their well-being (noting that not all students provided post-course data, *see above*). We also collected 30 testimonials from LSW students who later were chosen to be class facilitators. These responses were qualitatively coded (by two raters) and fell into two main themes: *Improvements* (i.e., ways in which the course improved well-being) and *Thoughts on the Course* (i.e., general thoughts on the content, design, and structure of the course). A full list of themes that appeared in >30% of the sample is provided in Table 1. The most frequent themes were as follows. For *Improvements*, students reported increased feelings of empowerment (70%); introspection of their emotional reaction (60%); and 53% intended to continue practicing the material outside of class. For *Thoughts on the Course*, students felt that the course material was highly applicable in everyday life (80%); that the course allowed for meaningful interactions with peers and the professor (70%); and that this class was overall extremely impactful (63%).

**Table 1:** Themes (and their descriptions) that appeared in >30% of the students’ testimonials, for *Improvements* (i.e., ways in which the course improved well-being) and *Thoughts on the Course* (i.e., general thoughts on the content, design, and structure of the course).

Themes	Description	%
*Improvements*		
1. Increased feelings of empowerment	Felt better equipped to handle future life challenges	70%
2. Increase in introspection	Learned the practice of examining and reflecting on emotional reactions	60%
3. Intention to continue with practices taught in class	Planned to keep utilizing mindfulness and compassion practices after the course	53%
4. Increase compassion for self and others	Gained a deeper appreciation for others and own emotional experiences	47%
5. Improved interpersonal relationship skills	Put communication and conflict resolution skills into practice in interpersonal relationships with a result of greater connection, understanding, and trust.	43%
6. Greater connection with community	Developed more connections with other students and felt a greater sense of connection within the UCSD community	40%
7. Awareness of personal values	Learned how to identify and act in line with one’s core values	40%
*Thoughts on Course*		
1. Highly applicable course material	Implemented lessons in everyday situations	80%
2. Meaningfully interactive class	Got opportunities to engage with other students and professor	70%
3. Extremely impactful class	Felt the course had stronger influence than other courses	63%
4. Timely class for college students	Expressed that the lessons were valuable to what was being experienced at this stage in life	47%
5. Desire for improved course accessibility	Expressed that the course would have been helpful at a prior time of life and expressed desire for their peers to also experience the course	40%
6. Promotion of relevant conversations outside of class	Course prompted discussion about the material with others outside class	37%
7. Inspired further engagement with mental health	Lessons expanded thinking beyond the curriculum leading to steps toward greater well-being (i.e., seeking therapy, practicing mindfulness, journaling, etc.)	33%

#### 3.2.2. Quantitative data showing improvements in student well-being

**Figure 1:**
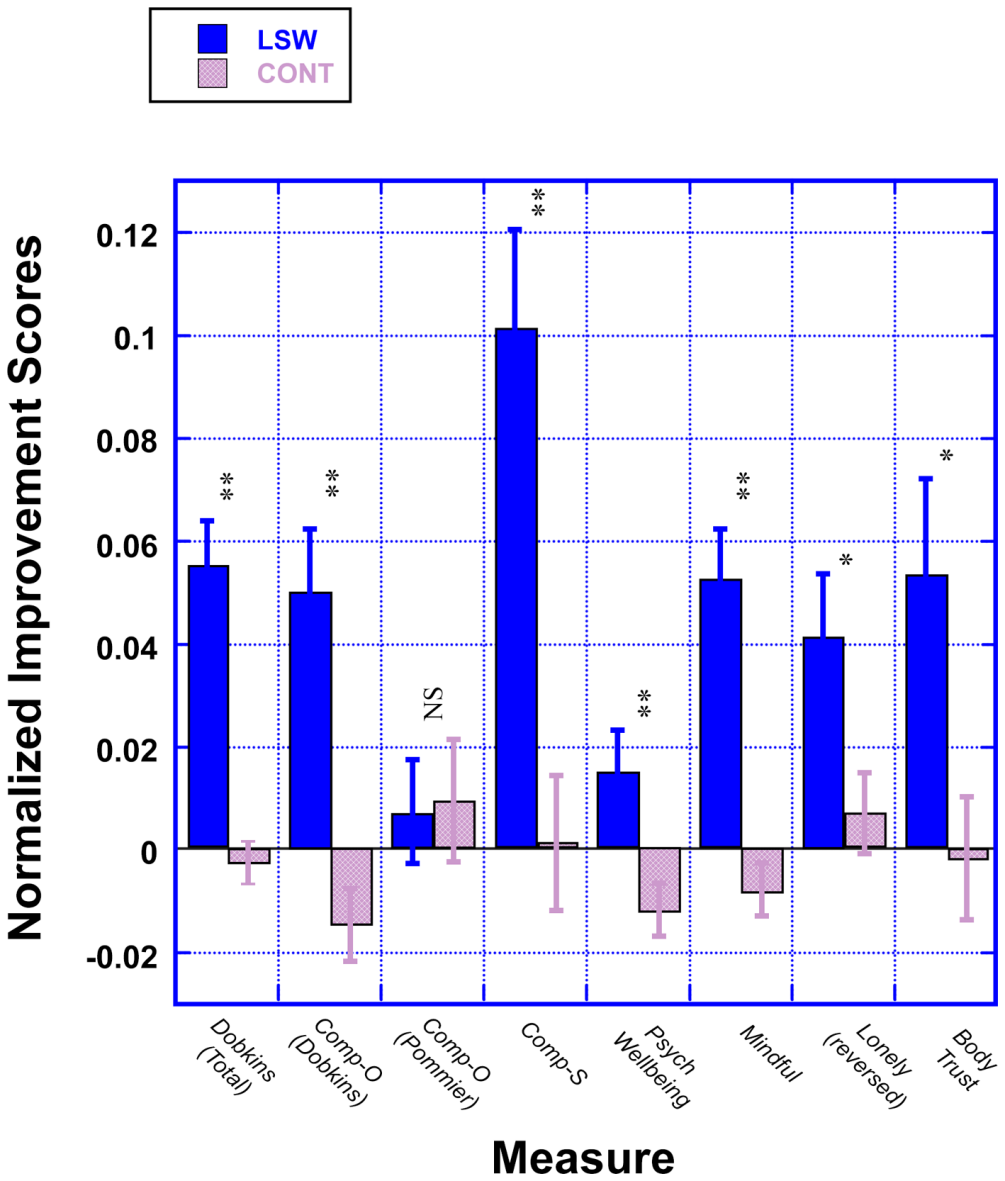
Improvement scores (and standard errors) are shown for LSW students (solid blue) and controls (hashed purple) for different constructs of self-reported well-being (**p < 0.001, *p < 0.01). Note that the improvement scores are “normed” by dividing the mean difference score by the total number of possible points for each measure, so that all measures can be presented, and compared, on the same plot. List of outcome measures: (1) Dobkins Scale: (A) Dobkins (Total): An in-house measure with 25 items that tests the main constructs of the LSW course. (B) Compassion for Others—Dobkins (“*Comp-O, Dobkins*”): 5 items from the Dobkins Scale. (2) Compassion for Others Scale—Pommier (“*Comp-O, Pommier”*): 16 items (24). (3) Self-Compassion Scale (“*Comp-S*”): 12 items (25). (4) Psychological Well-being (“*Psych Well-being*”): 18 items (26). (5) Five-Facet Mindfulness Questionnaire (“*Mindful*”): 20 items (27). (6) UCLA Loneliness Scale, V3 (“*Lonely*”): 20 items (28). (Reverse scored so that positive values reflect a *decrease* in loneliness). (7) Body Trust: 3 items from the MAIA Interoception Scale (29).

Mean improvement scores, calculated by subtracting pre-course from post-course data, are presented in Figure 1. Results are shown for seven different constructs of self-reported well-being, six of which were standardized measures. The seventh measure of well-being was developed to test the main constructs of the LSW course. In addition to presenting the total score data for this “in-house” measure, we present the data from one of its subscales (“Compassion for Others”) to help understand the insignificant result we observed for Pommier’s measure of this construct. The results of our analyses show that, with the exception of the Pommier scale, all improvement scores were significant, even after a Bonferroni correction (all *p-*values <0.007), with effective sizes ranging from small (0.27, for UCLA Loneliness) to large (0.75 for Self-Compassion, see Table 2 for full statistical results).

**Table 2:** Statistical results comparing improvement scores of students enrolled in LSW compared to control.

Outcome measures	LSW		Control		*t*	*df*	*p*	Cohen’s *d*
	*M*	*SD*	*M*	*SD*				
Dobkins (*Total*)	0.055	0.105	−0.003	0.062	6.470	353	<0.001	0.709
Compassion for Others (*Dobkins*)	0.050	0.135	−0.015	0.100	5.116	353	<0.001	0.561
Compassion for Others (*Pommier*)	0.007	0.090	0.009	0.093	−0.083	131	0.470	−0.014
Compassion for Self	0.101	0.168	0.001	0.099	4.310	131	<0.001	0.754
Psychological Well-being	0.015	0.089	−0.012	0.078	3.056	353	<0.001	0.335
Mindfulness	0.052	0.112	−0.008	0.079	5.861	353	<0.001	0.643
Loneliness (*reversed*)	0.041	0.141	0.007	0.114	2.499	353	0.006	0.274
Body Trust	0.053	0.215	−0.002	0.183	2.562	353	0.005	0.281

Note that the Compassion for Others (Dobkins) measure is a subscale of the Dobkins (Total) measure. Also note the Compassion for Others (Pommier) and Compassion for Self-scales have a smaller sample size than the other measures because we started collecting data for them later, in Spring 2021.

**Figure 2:**
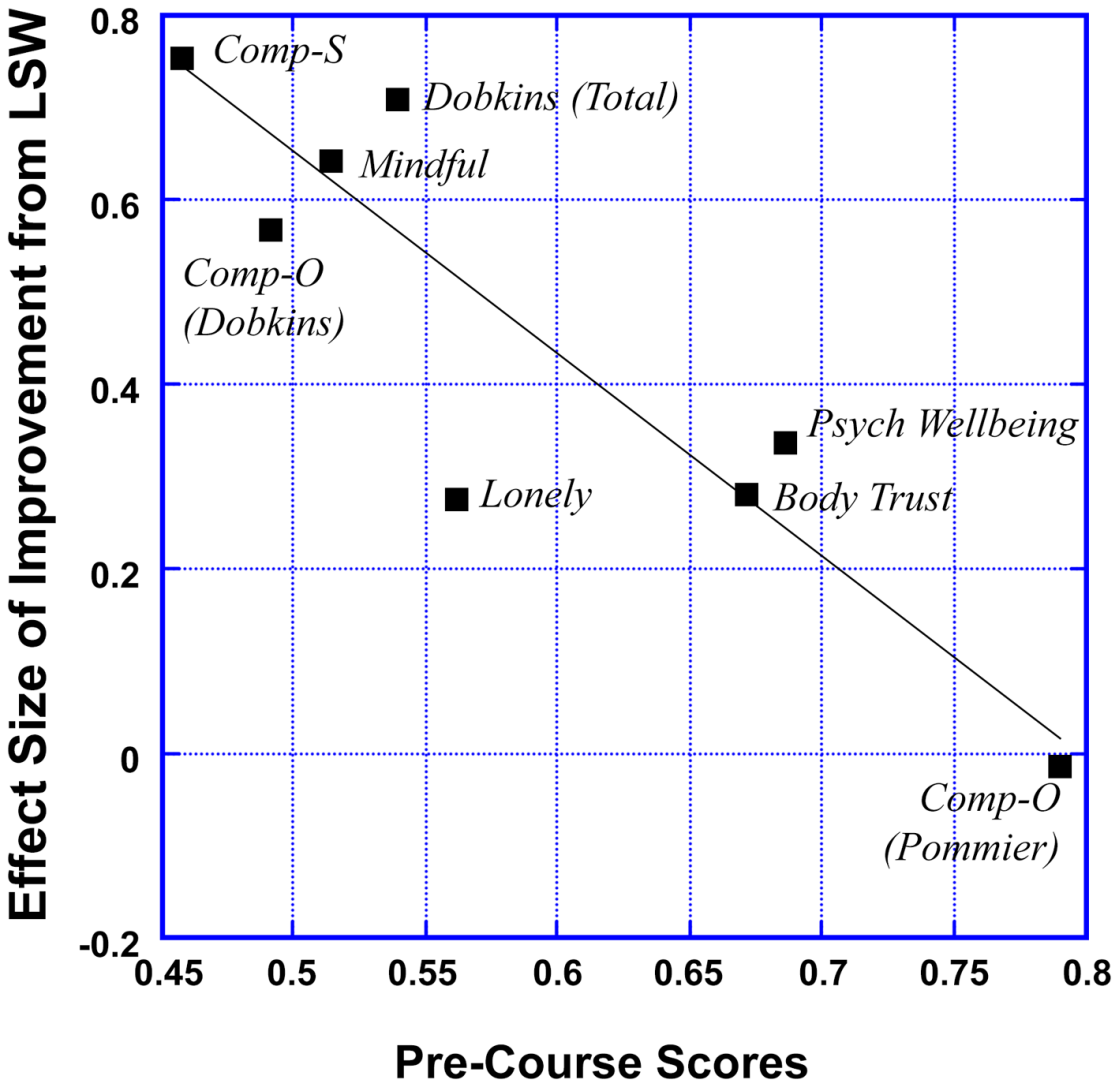
Scatter plot showing the strong negative relationship (*r* = −0.9) between mean pre-course scores and the effect size (Cohen’s *d*) of the improvement in well-being as a result of taking LSW across the different measures.

Because we were surprised by the insignificant result for the Pommier Compassion for Others scale, we conducted further investigation of all our measures. What became immediately obvious was that the measures that showed the largest effect sizes were the ones where participants started out low in the pre-course score, and this relationship had a large effect size with an *r*-value of −0.90 (see Figure 2). For example, on the Compassion for Others Scale (24), LSW students started out at 80% of the maximum possible on that measure (i.e., near ceiling), and this is the measure that showed the smallest (and non-significant) effect size when looking at improvement scores. For this reason, it is important to use measures where students are not starting out near ceiling (or near floor if the measure is a negative construct, like loneliness).

Upon further reflection, we are not surprised that students at our college are near ceiling on the Pommier et al. measure of Compassion for Others, as it taps into caring about other people’s suffering in situations where there is *no personal conflict* in helping someone in need. By contrast, in the LSW course, we specifically focus on what students (and people in general) really struggle with—which is how to be compassionate in *difficult situations*. We were therefore happy to see that the Compassion for Others subscale of the “Dobkins” measure—which focuses on compassion for difficult people/situations, showed significant (*p* < 0.001) and moderate (effect size = 0.56) improvement (see Figure 1). Thus, the LSW course reveals clear efficacy in improving compassion for others.

In a final analysis, we asked whether any of the observed effects of group (LSW vs. controls) differed by gender or the modality in which the class was taught. To this end, we conducted two-way ANOVAs for Gender (*Group*: LSW vs. Control × *Gender*: Female vs. Male) and Modality (*Group*: LSW vs. Control × *Modality*: In-Person vs. Zoom), for each of the well-being measures (16 total ANOVAs) and found no significant interactions (all *p* > 0.09). Although these null findings suggest that the beneficial effects of taking the LSW course did not vary across gender and modality, they should be interpreted with caution as our sample size is likely too small to observe interactions. This is particularly important for the Modality question; if it truly is the case that Zoom teaching is as effective as in-person, this could have important implications regarding scalability.

## 4. Practical implications and lessons learned

While many US colleges provide mental health services/resources, it is our belief that the most effective way to bring experiential well-being to students is to create for-credit Learning Sustainable Well-being (LSW) courses that provide the needed skills. As the Dalai Lama pointed out in his 2017 commencement speech at UC San Diego, colleges were once religious institutions, which provided *both* academic and spiritual guidance, but with the secularization of universities, that spiritual guidance is painfully missing. A solution is for colleges to implement a comprehensive system of guidance so that students can flourish academically and emotionally and be ready for an uncertain future that inevitably includes the ramifications of other societal crises, such as the climate crisis. Offering students LSW courses that focus on building resilience in the face of adversity can provide them the skills they need to deal with whatever future lies ahead.

The question we have grappled with is how best to create and scale up these LSW courses on college campuses. Should we take a *bottom-up* approach, recruiting other faculty to teach these LSW classes until there is a critical mass on campus, or a *top-down* approach, convincing the administration to oversee, and encourage participation in, an official LSW “program”? The top-down approach inevitably means meeting with top administrators, not only to get their “buy in,” but to figure out what administrative policy needs to change to make things happen. In our experience, we have found that the top-down and bottom-up approaches go hand in hand, which we experienced as follows.

In the Spring of 2019, we met with the Chancellor (and other top administrators) asking for their support of an official LSW program at our university. We argued that all students, even those who are not currently experiencing severe mental health concerns, are in need of a course that teaches them to have compassion for self and others. We also reported that the current LSW course was filling to capacity, with a waitlist, and that students were reporting that the course was “changing their lives.” Although administrators were sympathetic, they were clearly not signing on the bottom line. This seemed baffling at first; why would not the administration adopt this “no-brainer” idea? Over the next few years, it started to become clear that what might win them over would be to provide evidence that (1) the course improves student well-being (which we now have, *see above*) and (2) a sufficient number of faculty could be recruited to teach an LSW course.

Our bottom-up approach for faculty recruitment involved asking various faculty if they would be willing to teach our already-created curriculum (as opposed to creating their own well-being curriculum). We thought this “adopt our curriculum” approach would be best as it would ensure some quality assurance as well as make it easier to measure the efficacy of the course in improving well-being across a diverse sample of faculty. Because we already had two of us (the first and second authors) in the Psychology department successfully teaching the LSW course, in 2021, we started asking our psychology colleagues if they would join in. Although many were sympathetic to our cause, we were met with two obstacles. *First*, some faculty members reported not feeling well equipped to teach an experiential LSW course, having never themselves adopted a well-being practice. We believe this obstacle can be overcome by creating/offering workshops in which faculty get trained to teach the LSW course during the summer months. The goal is to secure funds to pay them for their training time, noting that, in addition to the financial benefit, partaking faculty should experience a psychological benefit of learning the course material- just like the students who take the course!

A *second* obstacle we experienced concerns teaching load; many faculty reported that they did not have enough time in their schedule to take on another class. To address this obstacle, we spent a lot of time with departmental and university-level administrators to discuss potential solutions: Could the class be converted to a 2- or 4-unit (rather than the current 1-unit) class? How best could the course fulfill student requirements for graduating? Could the experiential spirit of the course be maintained if it were changed to a 2- or 4-unit course, which would then require a letter grade? Could faculty teach the LSW course by being offered release from another course? After much deliberation, we decided it best to keep the LSW course as a 1-unit P/NP, taught above current teaching load and compensated with $1 K (that can be applied toward the faculty’s research funds).

Once these obstacles seemed sufficiently resolved, we then launched our bottom-up campaign to recruit faculty from *other* departments on campus. In Winter 2023, we reached out to the chairs of several departments on campus, asking if they would circulate a recruitment letter to their faculty. To our pleasant surprise, all the chairs agreed, and within a few weeks, we had a coalition of 6 faculty interested in getting trained in and teaching the LSW course (from Biology, Cognitive Science, History, Sociology and Political Science). All these faculty members immediately recognized the need for such courses at our university, which was quite encouraging.

Now that we have a growing coalition of faculty who are interested in teaching LSW, as well data showing the efficacy of the LSW course, we are resuming discussions with the administration, in the hope that all of our bottom-up achievements will be met with top-down endorsement of an official “LSW program.” This stamp of approval from the administration will make it much easier to: (1) get the necessary resources to recruit LSW faculty, (2) expedite the approval of these LSW courses in different departments, (3) document the implementation of the LSW course across departments (e.g., asking well it works for faculty from different disciplines to teach the course through their own lens), (4) fund the collection and analysis of data investigating the efficacy of the LSW course in different departments, and (5) bridge the cultural gap between faculty and students, thereby building campus community.

On a final note, we end with a call to action for all college administrators. Institutions like to position themselves as preparing young people for the future, however, they are currently neglecting some of the most crucial tools needed to be human: empathy, compassion, resilience, even listening. To make matters worse, we are sending them out into a world of colliding crises, which they are not equipped to tackle. The idea of for-credit LSW courses offers a creative solution, and it is up to the administration to acknowledge this, to act as if we are in crisis mode. Because we are.

## Data availability statement

The raw data supporting the conclusions of this article will be made available by the authors, without undue reservation.

## Ethics statement

The studies involving human participants were reviewed and approved by the University of California, San Diego Office of IRB Administration. The patients/participants provided their written informed consent to participate in this study.

## Author contributions

KD conceived of the idea. KD, JD, and DL wrote the manuscript together. KD and TB analyzed the quantitative data. All authors contributed to the article and approved the submitted version.

## Funding

This work was supported by the *T Denny Sanford Institute for Empathy and Compassion* under the 1-year pilot study to scale up the LSW course.

## Conflict of interest

The authors declare that the research was conducted in the absence of any commercial or financial relationships that could be construed as a potential conflict of interest.

## Publisher’s note

All claims expressed in this article are solely those of the authors and do not necessarily represent those of their affiliated organizations, or those of the publisher, the editors and the reviewers. Any product that may be evaluated in this article, or claim that may be made by its manufacturer, is not guaranteed or endorsed by the publisher.
